# The Relationship between Post-Traumatic Symptoms, Parenting Style, and Resilience among Adolescents in Liaoning, China: A Cross-Sectional Study

**DOI:** 10.1371/journal.pone.0141102

**Published:** 2015-10-21

**Authors:** Yanxue Zhai, Kun Liu, Lin Zhang, Han Gao, Zhuo Chen, Siyi Du, Lili Zhang, Yu Guo

**Affiliations:** 1 College of Nursing, Liaoning Medical University, Jinzhou, Liaoning, People’s Republic of China; 2 College of Basic Medical Sciences, Liaoning Medical University, Jinzhou, Liaoning, People’s Republic of China; Central Institute of Mental Health, GERMANY

## Abstract

**Background:**

In China, a growing number of adolescents have experienced traumatic events that have resulted in PTSD (post-traumatic stress disorder). Post-traumatic symptoms are common psychological problems in adolescents who have experienced traumatic events. However, existing studies tend to focus on the factors influencing PTSD, such as the response styles and social support, and studies on the relationships between parenting style, resilience and post-traumatic symptoms are still rare.

**Objectives:**

To analyze the relationships between parenting style, resilience and post-traumatic symptoms among adolescents in China.

**Methods:**

A cross-sectional survey was conducted from June to December 2013 in the Liaoning Province, China. N = 5765 adolescents (aged 12 to 18 years old) were ultimately chosen to participate. The Chinese version of the Essen Trauma Inventory for Kids and Juveniles (ETI-KJ), a modified version of the Parental Authority Questionnaire, and the Chinese Resilience Scale were used to estimate the post-traumatic symptoms, parenting style, and resilience, respectively. Pearson’s correlations, multiple linear regression analyses and structural equation modeling (SEM) were applied to analyze the data.

**Results:**

Of the adolescents, 39.76% (N = 2292) had been exposed to traumatic events during their lives. The prevalence of probable PTSD at the time of the interview (one-month-prevalence) was 12.65%. Parenting style and resilience were significantly associated with post-traumatic symptoms. According to the SEM, parenting style had a significant direct effect on resilience (0.70, P<0.01) and post-traumatic symptoms (-0.15, P<0.05), and resilience had a significant direct effect on the post-traumatic symptoms (-0.43, P<0.01). Furthermore, parenting style had a significant indirect effect (-0.43×0.70 = -0.30. P<0.01) on the post-traumatic symptoms through resilience. The SEM significantly explained 49% of the variance in resilience and 30% of the variance in post-traumatic symptoms.

**Conclusions:**

Parenting style and resilience have significant effects on adolescents’ post-traumatic symptoms. Schools and social-related departments could share knowledge on the impact of parenting style with parents enabling them to improve their own parenting style and their children’s resilience and ability to respond effectively to traumatic events.

## Introduction

PTSD is a commonly occurring disorder that often has a duration of many years and it is frequently associated with exposure to multiple traumatic events [1]. In school-aged children in Palestine, 54.7% have experienced at least one traumatic event in their lifetime, and the prevalence of PTSD is 34.1% [2]. The prevalence of PTSD among young people (14–24 years old) in Germany is 1.5% [3], and it is 8.08% [4] among adolescents (12–17 years old) in America. In China, an increasing number of publications focused on PTSD in adolescents after the 2008 Sichuan earthquake because adolescents tend to be more vulnerable. According to one investigation [5], five months after the earthquake, the prevalence of PTSD in adolescents in the earthquake districts was 46.61% and decreased to 13.3% at 18 months after the earthquake. Substantial research has focused on adolescents in the Sichuan Province, but much less research has been performed on other regions in China. Not only can natural disasters cause PTSD, other life-threatening events (wars, serious accidents, severe diseases, physical abuse, etc.) could also lead to PTSD. Therefore, research on adolescents in the regions outside of the earthquake districts is also important. PTSD has become an increasing public health problem worldwide. Hence, systematic community and population-based studies should be advocated to investigate the severity and process of post-traumatic reactions among adolescents [6–7].

Resilience is a psychological function whereby individuals recover after experiencing adversity or traumatic events using a successful response to a self-adjusting mechanism [8–9]. It is an effective protective factor when people experience detrimental events [10]. Research in the United States has shown that resilience has a significant negative effect on post-traumatic symptoms [11]. Moreover, it was revealed that, because of their resilience, many individuals do not have mental disorders despite their exposure to traumatic events [12]. Furthermore, there have been many studies on the influence of resilience and its effect on mental health; these studies have drawn many valuable conclusions, such as that resiliency has many protective factors, including a good parenting style.

In the 1990s, researchers studied the effects of the family environment on child development by observing parent-child interactions. They proposed that parental behaviors influence children’s development through parenting styles. Baumrind noted that parenting styles could be divided into the following three types: permissive, authoritarian, and authoritative [13]. It is important to emphasize that the authoritative parenting style is warm and democratic, whereas the authoritarian parenting style is restrictive and hostile [14], and permissive parenting is characterized by very few rules or restrictions [15]. The studies on resilience and parenting style have indicated that an authoritative parenting style has a significant positive effect on resilience, while the effects of the authoritarian parenting and permissive parenting styles on resilience are not significant [16]. Moreover, one study found that the authoritarian parenting style is an important factor that influences physical punishment in families [17]. In authoritarian parenting, physical or mental abuse may be related to post-traumatic symptoms in children.

According to the aforementioned studies, resilience is related to post-traumatic symptoms, and parenting style is related to resilience. Therefore, we hypothesized that parenting style and resilience may be associated with post-traumatic symptoms. The aim of this paper is to investigate the degree of post-traumatic symptoms among adolescents in the Liaoning Province, China, and to estimate the synthesized relationship between parenting style, resilience and post-traumatic symptoms.

## Method

### Participants and data collection

A cross-sectional study was conducted in the Liaoning province. This investigation adopted a multistage stratified cluster random sampling method. Liaoning province includes 14 cities and is located in northeast China. According to the GDP ranking of Liaoning province in 2012, Shenyang city and Dalian city represent the highest level of socioeconomic development, Yingkou city and Jinzhou city represent moderate levels of socioeconomic development, and Liaoyang city and Tieling city represent the lowest level of socioeconomic development. For this study, 3 primary schools and 3 middle schools representing the average teaching and economic level of each city were randomly selected from each city. The students attending the 36 schools included in this study were considered to be representative of all adolescents in Liaoning province. Finally, we selected one entire class from each selected primary and middle school. In China, children in primary school grades 1–2 are too young to complete questionnaires and, as a result, they were excluded from the study.

On the day before the questionnaires were distributed, with the agreement of the Liaoning Medical University ethics committee and the approval and assistance of the head teachers, we issued informed consent forms to each student to give to their parents. If the parents and students were willing to participate in the investigation, they wrote “agree” on the informed consent forms. The next day, we distributed our questionnaires to the students who had agreed to participate. First, we performed a preliminary investigation in Jinzhou city in May 2013. We randomly selected one primary and one middle school and issued questionnaires to the students who agreed to participate in our investigation. Five hundred thirty-five students participated, and 500 students responded effectively. Then, according to the multistage stratified cluster random sampling described above, we performed the community study from June to December 2013. We invited 6,500 students (aged 12 to 18 years old) to participate in this investigation; then, a total of 6,225 students agreed to participate in the study, and 5,958 responded (95.71% response rate); of these, 5,765 valid questionnaires were ultimately acquired (96.76% effective response rate).

Among those who responded effectively, 39.76% (N = 2292) had experienced traumatic events during their lifetime. One thousand one hundred two (48.08%) of the adolescent participants were male, and 1190 (51.92%) of the participants were female. The mean age of the adolescents was 12.45 years old (*SD* = 1.54 years). The number of adolescents per grade was 518 (22.60%) in sixth grade, 536 (23.39%) in seventh grade, 694 (30.28%) in eighth grade, and 544 (23.73%) in ninth grade. One thousand sixty-one (46.29%) of the adolescents were in a student cadre. Moreover, 914 (39.88%) of the adolescents were in inharmonious families, while 1378 (60.12%) of the adolescents were in harmonious families.

### Measuring instruments

The questionnaire had four parts, and Part 1 collected the following sociodemographic information: gender, grade, age, student cadre or not, and family status. It was explained that family status is the state in which family members get along. We divided the family status into inharmonious and harmonious families. The harmonious family status is a state in which the family members get along well. On the other hand, the inharmonious family status involves quarreling and scolding children. Participants could subjectively choose their family status, harmonious family or inharmonious family.

Part 2 was the Chinese version of the Essen Trauma Inventory for Kids and Juveniles (ETI-KJ), compiled by Tagay et al. [18], which was used to assess the post-traumatic symptoms in the participants. The scale has 5 sections that have been well validated in China [19], especially with teens aged 12 to 17 who are screened for mental disorders after trauma. Section 1 of the ETI-KJ asks about the respondent’s life experience of traumatic events using a list of 12 potential trauma and loss events. The subjects were asked whether they had experienced any traumatic events, including child abuse and negligence, violence, traffic accidents, and natural disasters, either themselves or as described to them by witnesses. The 13^th^ item asks respondents to identify the worst event they ever experienced from among the 12. If the student’s worst event was not included in one of the 12 scale items, he or she could write it in. Then, to prompt the subjects, the subsequent questions were related to their most severe traumatic events. Based on how the students described the worst aspects of the traumatic events, section 2 investigated the seriousness of the events using objective descriptions and subjective assessments that correspond to the DSM-IV diagnosis criteria for acute stress disorder (ASD) and PTSD. Section 3 asked about the students’ current (in the past four weeks) post-traumatic symptoms. The 23 items asked about intrusion (5 items), avoidance (7 items), hyper-arousal (5 items) and dissociation (6 items) rated on a 4-point Likert scale that ranged from “does not match at all” (0 points) to “completely matches” (3 points). The 24^th^ item asked about possible somatic symptoms as well as the post-traumatic symptoms. Section 4 asked about when the post-traumatic symptoms began, how long they lasted and the pain caused by the traumatic events, using a 6-point Likert scale that ranged from “no pain” (0 points) to “extremely painful” (5 points). Looking at the interpersonal relationships, ability to learn, and hobbies, section 5 asked about the effects of post-traumatic symptoms on the students’ daily lives using a 4-point Likert scale that ranged from “no effect” (0 points) to “serious effect” (3 points).

The assessment of the post-traumatic symptoms was based on the total scores for the 23 items in section 3; the higher the score, the more serious the individual’s post-traumatic symptoms. According to the study by Tagay, on the premise of the 12 potential trauma events in section 1 meeting the DSM-IV A criteria 1 and 2, scores on the three dimensions of intrusion, avoidance and hyper-arousal that total more than 16 points are considered indicative of probable PTSD. Total scores over 35 on the four dimensions of intrusion, avoidance, hyper-arousal and dissociation are considered to indicate probable ASD.

Section 1 of the ETI-KJ was used to screen those adolescents who had experienced at least one traumatic event, and section 3 assessed their post-traumatic symptoms. A study conducted in China showed that this questionnaire had good reliability (Cronbach’s α coefficient is 0.918) and validity [19]. Using the method of principal components, the following four factors were extracted using exploratory factor analysis (EFA) of the pilot sample’s scores for section 3 of the ETI-KJ: intrusion (5 items), avoidance (7 items), hyper-arousal (5 items) and dissociation (6 items). Then, in the formal sample, we performed confirmatory factory analysis (CFA) of the instrument and attained a four-factor structure, Model T, that was supported by the investigation data with the following: GFI (goodness of fit index), NFI (normed fit index), CFI (comparative fit index), IFI (incremental fit index) >0.9, and RMSEA (root of the resulting ratio gives the population) = 0.00, lambda x (the factor-loading parameters) >0.67. The Cronbach’s α coefficients of internal consistency were 0.842, 0.703, 0.752 and 0.728 for intrusion, avoidance, hyper-arousal and dissociation, respectively. These results indicate good construct validity and acceptable reliability.

Part 3 of our questionnaire used a modified version of the Parental Authority Questionnaire (PAQ) to assess the parenting style (e.g., “my parents tell me how I should act and explain the reasons why; my parents feel that parents must use force to get children to act the way they supposed to; my parents feel that children can do whatever they like”) [20]. The original PAQ consists of 30 items, and there were 10 items per parenting style (authoritarian, authoritative, and permissive). Trinkner et al. shortened the scale by selecting 4 items for each parenting style and by using a four-point Likert scale (1: disagree strongly; 4: agree strongly) [21]. Studies in China showed that the modified version of the PAQ had accepted validity and reliability (the Cronbach’s α coefficients of the three subscales are 0.63, 0.61, and 0.41, respectively) [22]. The following three factors were extracted using an EFA of the modified PAQ in the pilot sample: authoritarian, authoritative, and permissive parenting. In the formal sample, a CFA of the instrument revealed a three-factor structure Model P that was supported by the data (GFI, NFI, CFI, IFI >0.9, and RMSEA = 0.053). The Cronbach’s α coefficients of internal consistency were 0.867, 0.841 and 0.886 for the authoritarian, authoritative and permissive parenting styles, respectively. These results indicate good construct validity and reliability.

Part 4 utilized the Chinese Resilience Scale to evaluate the students’ resilience (e.g., “failure always makes me feel discouraged; I feel more experienced after suffering from setbacks; when I am met with difficulties, I do not know how to address them; and parents lack confidence and mental support for me”). It was designed by Yueqin et al. [23] for Chinese adolescents at the Peking University Department of Psychology, and it was verified as valid. The scale consists of 27 items that were classified into five dimensions. This questionnaire was widely used in China and has good reliability and validity. An EFA of this questionnaire extracted the following five factors: goal planning, help-seeking, family support, affect control, and positive thinking. In the formal sample, a CFA of the instrument showed that a five-factor Model R was supported by the data (GFI, NFI, CFI, IFI >0.9, and RMSEA = 0.073); the Cronbach’s α coefficients of internal consistency were 0.731, 0.804, 0.932, 0.844, and 0.862 for goal planning, help-seeking, family support, affect control and positive thinking, respectively. These results also indicate good construct validity and reliability.

### Statistical analysis

**Fig 1:**
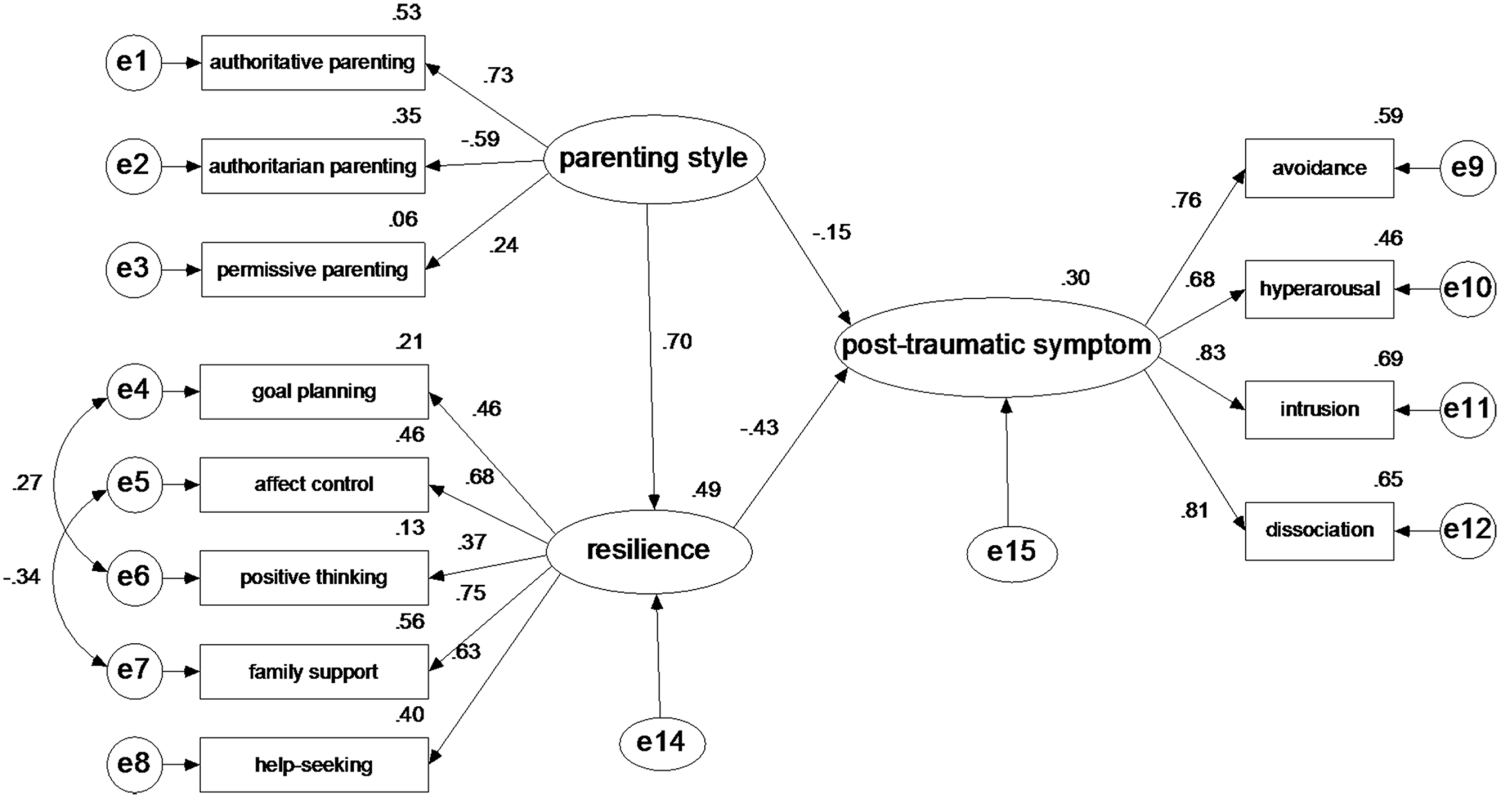
The structural equation model on the relationships between parenting style, resilience and post-traumatic symptoms in the total sample data. e1—e12: the measurement error of each observed variable to estimate latent variables. e14—e15: the residual that may affect the endogenous latent variables except the exogenous latent variables.

SPSS 17.0 and AMOS 17.0 were used for the statistical analyses. First, the descriptive analyses of the sociodemographic variables and types of traumatic events assessed were executed. Then, Pearson’s correlation coefficients were calculated to assess the relationships between the parenting style, resilience and post-traumatic symptoms. In addition, multiple linear regression was performed using the post-traumatic symptom score as the outcome variable and parenting style and resilience as continuous independent variables, controlling for age, gender, grade, student cadre, and family status. Finally, structural equation modeling (SEM) was applied to further verify the hypothetical relationships between several latent variables (“parenting style”, “resilience” and “post-traumatic symptoms”), and the process of parameter estimations could exclude measurement error. According to related research and theories, the hypothetical relationships model was created and is shown in Fig 1. Three measurement models were presented as follows: parenting style as an exogenous latent variable affecting three exogenous observed variables (authoritative parenting, authoritarian parenting and permissive parenting) with respective measurement errors (e1-e3); resilience as an endogenous latent variable affecting five endogenous latent variables (goal planning, affect control, positive thinking, family support, and help-seeking) with respective measurement errors (e4-e8); post-traumatic symptom as ultimate endogenous latent variables affecting four endogenously observed variables (avoidance, hyperarousal, intrusion and dissociation) with respective measurement errors (e9-e12). The structural path hypothesis is that the parenting style has direct and indirect effects through resilience with an unexplained disturbance (e14) on post-traumatic symptoms with an unexplained disturbance (e15). Furthermore, Table 1 shows the results of goodness-of-fit indexes of SEM on the total sample and individual samples. Although there was little difference between these single samples, the results could be accepted.

**Table 1:** Goodness-of-fit indexes of SEM on different samples.

Model	*N*	*χ* ^*2*^ */df*	GFI	AGFI	NFI	IFI	CFI	RMSEA
**Experience value**	^___^	^____^	>0.90	>0.90	>0.90	>0.90	>0.90	<0.08
**Model T (Total Sample)**	2292	8.368	0.958	0.934	0.835	0.852	0.850	0.057
**Model S1 (Sample 1)**	562	3.455	0.950	0.920	0.759	0.816	0.811	0.066
**Model S2 (Sample 2)**	1730	7.373	0.956	0.930	0.831	0.850	0.849	0.061
**Model S3 (Sample 3)**	914	4.943	0.949	0.919	0.802	0.835	0.833	0.066
**Model S4 (Sample 4)**	1378	5.699	0.959	0.935	0.831	0.856	0.855	0.058

Sample 1: adolescents who experienced traumatic events that were dependent on their parents.

Sample 2: adolescents who experienced traumatic events that were independent of parents.

Sample 3: adolescents who are in an inharmonious family.

Sample 4: adolescents who are in a harmonious family.

GFI: goodness of fit index; AGFI: adjusted goodness of fit index; NFI: normed fit index; IFI: incremental fit index; CFI: comparative fit index; RMSEA: root of the resulting ratio gives the population.

## Results

### The description of probable PTSD among adolescents

Of the adolescents in this study, 39.76% (N = 2292) had experienced traumatic events during their lifetime. The one-month prevalence of probable PTSD (total scores on the three dimensions of intrusion, avoidance and hyper-arousal of more than 16 points) was 12.65% at the time of assessment. Of these 2292 participants, 312 (5.41% of the total participants) adolescents were from Shenyang city, and among them, 95 (1.65% of the total participants) adolescents were considered to suffer from probable PTSD. Three hundred eighteen (5.52%) students were from Dalian city, and among them, 95 (1.65%) adolescents were considered to suffer from probable PTSD. Three hundred forty-two (5.93%) students were from Jinzhou city, and among this sample, 96 (1.67%) adolescents were considered to suffer from probable PTSD. In Yingkou city 390 (6.76%) adolescents had experienced a traumatic event, and among them, 139 (2.41%) adolescents were considered to have probable PTSD. Four hundred fifty-seven (7.93%) adolescents were from Liaoyang city, and among them, 137 (2.38%) adolescents were considered to exhibit probable PTSD. Finally, 473 (8.20%) students were from Tieling city, and among them, 167 (2.90%) adolescents were considered to suffer from probable PTSD.

Table 2 shows the types and prevalence of each traumatic event. In addition, to assess whether the traumatic events are independent of or involve parents, we counted the cases with traumatic events that were and were not independent of the parents; they were 1,730 (30.01% of all participants) and 562 (9.75% of all participants), respectively.

**Table 2:** The frequency of different types of traumatic events that adolescents experienced.

	Type of traumatic event	NO. (%)
1	Natural disaster (e.g., earthquake, flood, thunderstorm)	421 (7.30)
2	Serious accidents, fire accidents or explosions (e.g., traffic accident)	750 (12.79)
3	Severe diseases (e.g., cancer, heart disease, major surgery)	349 (6.05)
4	Violent attacks by strangers (e.g., criminal assault, robbery)	144 (2.50)
5	Violent attacks by relatives or acquaintances (e.g., criminal assault, robbery)	179 (3.10)
6	One person died who is very important to you	797 (13.82)
7	Was placed in chains	39 (0.68)
8	Suffered from sexual assault by strangers (e.g., forced sexual contact, rape)	23 (0.40)
9	Suffered from sexual assault by relatives or acquaintances (e.g., forced sexual contact, rape)	16 (0.28)
10	Resided in a war zone	25 (0.43)
11	Neglected, abandoned (e.g., often rejected, rarely gets attention from parents)	236 (4.09)
12	Other things that cause mental burden (e.g., being away from their parents or family or their parents divorcing)	265 (4.60)
Traumatic events dependent on their parents (No. 5, 9, 11, and 12)		562 (9.75)
Traumatic events independent of their parents (No.1, 2, 3, 4, 6, 7, 8, and 10)		1,730 (30.01)

### The correlativity of post-traumatic symptoms, parenting style and resilience

The associations between the post-traumatic symptoms, parenting style and resilience in adolescents who went through traumatic events that were or were not independent of their parents may be different. Therefore, we performed a correlation analysis in different adolescent samples. Sample 1 (N = 562) consisted of adolescents who went through traumatic events that were dependent on their parents, while Sample 2 (N = 1730) consisted of adolescents who went through traumatic events that were independent of their parents. Table 3 shows that the adolescents’ post-traumatic symptoms were significantly and negatively related to the five subscales of resilience and authoritative parenting, but they were positively related to authoritarian parenting in all samples. To understand the associations between the parenting style and resilience, we performed a correlation analysis on the total sample. Table 4 shows that authoritative parenting was significantly and positively related to the five subscales of resilience, and authoritarian parenting was significantly and negatively related to the five subscales of resilience. In addition, permissive parenting was significantly and positively related to the following three subscales of resilience: “goal planning”, “positive thinking” and “family support”.

**Table 3:** Correlation between parenting style, resilience and post-traumatic symptoms in different samples.

		Post-traumatic symptoms of sample 1 (N = 562)	Post-traumatic symptoms of sample 2 (N = 1730)	Post-traumatic symptoms of all data (N = 2292)
**Parenting style**	**authoritative**	-0.202**	-0.166**	-0.210**
	**authoritarian**	0.218**	0.225**	0.257**
	**permissive**	0.032	0.037	0.023
**Resilience**	**goal planning**	-0.140**	-0.170**	-0.192**
	**affect control**	-0.263**	-0.342**	-0.342**
	**positive thinking**	-0.076	-0.080**	-0.098**
	**family support**	-0.177**	-0.234**	-0.256**
	**help-seeking**	-0.158**	-0.276**	-0.273**

**The correlation is significant at the 0.01 level (2-tailed).

Sample 1: consists of adolescents who experienced traumatic events that were dependent on their parents.

Sample 2: consists of adolescents who experienced traumatic events that were independent of their parents.

**Table 4:** Correlation between parenting style and resilience.

	Goal planning	Affect control	Positive thinking	Family support	Help-seeking	Total resilience score
**Authoritative parenting**	0.357**	0.215**	0.296**	0.461**	0.287**	0.463**
**Authoritarian parenting**	-0.134**	-0.231**	-0.088**	-0.330**	-0.212**	-0.302**
**Permissive parenting**	0.174**	0.014	0.179**	0.202**	0.019	0.148**
**Total parenting style score**	0.236**	0.002	0.228**	0.197**	0.062**	0.187**

** The correlation is significant at the 0.01 level (2-tailed).

With post-traumatic symptoms as the explained variable and sociodemographic characteristics as the controlled variables, the parenting style and resilience dimensions were taken as principal significant variables by a thrice repeated multiple linear regression analysis. According to Model R_1_ in Table 5, the variables of grade, family status and student cadre significantly contributed to post-traumatic symptoms, and a total of 0.6% of the criterion variance was explained (F = 3.921,P<0.01). On the basis of Model R_1_, multiple linear analysis in Model R_2_ showed that authoritative, authoritarian and permissive parenting styles were significantly associated with post-traumatic symptoms, and a total of 9.1% of the criterion variance was explained (F = 29.458, p<0.01). Model R_3_ was run with the resilience dimensions added based on Model R_2_, and three subscales of parenting style and four subscales of resilience (goal planning, affect control, family support and help-seeking) were identified as significant relevant variables of post-traumatic symptoms.

**Table 5:** Results of the multiple linear regression analysis by building progressive models with the post-traumatic symptoms as the dependent variable.

Explanatory Variables		^※^Model R_1_		^※^Model R_2_		^※^Model R_3_	
		t	P value	t	P value	t	P value
**Sociodemographic variables**	**gender**	-0.448	0.654	0.639	0.523	1.110	0.267
	**grade**	2.803	0.005	2.573	0.010	1.513	0.131
	**age**	-0.575	0.565	-0.149	0.882	0.057	0.954
	**family status**	-2.480	0.013	-3.068	0.002	-3.675	0.000
	**student cadre or no**	-2.107	0.035	-0.776	0.438	1.731	0.084
**Parenting style (b, 95%CI)**	**authoritative**	^____^	^____^	-6.993	0.000	-2.130	0.033
		^____^	^____^	-0.744(-0.953, -0.535)		-0.237(-0.454, -0.019)	
	**authoritarian**	^____^	^____^	9.694	0.000	6.544	0.000
		^____^	^____^	1.015(0.810, 1.220)		0.673(0.471, 0.875)	
	**permissive**	^____^	^____^	3.049	0.002	3.260	0.001
		^____^	^____^	0.365(0.130, 0.600)		0.375(0.150, 0.601)	
**Resilience (b, 95%CI)**	**goal planning**	^____^	^____^	^____^	^____^	-3.602	0.000
		^____^	^____^	^____^	^____^	-0.280(-0.433, -0.128)	
	**affect control**	^____^	^____^	^____^	^____^	-10.434	0.000
		^____^	^____^	^____^	^____^	-0.582(-0.692, -0.473)	
	**positive thinking**	^____^	^____^	^____^	^____^	1.660	0.097
		^____^	^____^	^____^	^____^	0.155(-0.028, 0.338)	
	**family support**	^____^	^____^	^____^	^____^	-4.169	0.000
		^____^	^____^	^____^	^____^	-0.374(-0.550, -0.198)	
	**help-seeking**	^____^	^____^	^____^	^____^	-3.458	0.001
		^____^	^____^	^____^	^____^	-0.192(-0.301, -0.083)	
**F value**		3.921		29.458		40.206	
**P value**		0.002		0.000		0.000	
**Adjusted R** ^**2**^		0.006		0.091		0.184	

^※^: Model R_1_: multiple linear regression model with post-traumatic symptoms as the dependent variable and sociodemographic variables as the independent variables.

Model R_2_: multiple linear regression model with post-traumatic symptoms as the dependent variable and sociodemographic variables and parenting style as the independent variables.

Model R_3_: multiple linear regression model with post-traumatic symptoms as the dependent variable and sociodemographic variables, parenting style and resilience as the independent variables.

### The synthetic relationship between the post-traumatic symptoms, parenting style and resilience

To identify a synthetic association between the post-traumatic symptoms, resilience and parenting style, we constructed the SEM with different samples. Fig 1 shows the standardized regression weights and squared multiple correlations of the SEM with post-traumatic symptoms and resilience as endogenous latent variables and parenting style as an exogenous latent variable on the total sample data (N = 2292). The three factor-loading parameters (0.24–0.73) in the parenting style matrix, the five factor-loading parameters (0.37–0.75) in the resilience matrix and the four factor-loading parameters (0.68–0.83) in the post-traumatic symptoms matrix reached significance (P<0.01). According to the structural path model, the parenting style had a significant direct effect on resilience (0.70, P<0.01) and post-traumatic symptoms (-0.15, P<0.05), and resilience had a significant direct effect on the post-traumatic symptoms (-0.43, P<0.01). Furthermore, the parenting style had a significant, indirect effect (-0.43×0.70 = -0.30. P<0.01) on the post-traumatic symptoms through resilience. Parenting style had a total impact of -0.45 on the post-traumatic symptoms and an impact of -0.30 that was produced by resilience as an intermediary variable. The SEM significantly explained 49% of the variance in resilience and 30% of the variance in post-traumatic symptoms, indicating that an additional 11.6% of the variance in post-traumatic symptoms was explained by the parenting style and resilience, excluding the measurement errors of the observed variables. Based on the modification indexes (MI), two measurement residual paths and the covariance between e4 and e6 (MI = 61.70) and between e5 and e7 (MI = 54.73) were recommended for inclusion.

Table 1 shows that the modified goodness-of-fit indexes of the SEM were accepted according to a previous study [24]. Considering the differences between the adolescents who went through traumatic events that were or were not independent of parents, and the differences between adolescents who were in an inharmonious or harmonious family, we conducted a structural equation model with different single samples to confirm whether the relationship in the SEM was or was not suitable. Then, the modified goodness-of-fit indexes of the SEM on the data from sample 1 (*N* = 562, consisted of adolescents who went through traumatic events that were dependent on their parents), sample 2 (*N* = 1730, consisted of adolescents who went through traumatic events that were independent of their parents), sample 3 (*N* = 914, consisted of adolescents who were in an inharmonious relationship with their parents) and sample 4 (*N* = 1378, consisted of adolescents who were in a harmonious relationship with their parents) are included in Table 1.

## Discussion

This study revealed that the prevalence of probable PTSD among adolescents at the time of interview (one-month-prevalence) was 12.65%, which was in line with another study (12.2% of all participants) investigated in Shanghai, China [25]. With respect to the types of traumatic events in Table 2, the prevalence of event 6 (one person died who is very important to you) is the highest, at 13.82%, while event 9 (suffered from sexual assault of strangers) is the lowest, at 0.40%. There are probably many complicated reasons that the prevalence of probable PTSD in China is higher than that in Europe or the United States. This may be due to the differences in the national conditions, life environment, parenting style and type of traumatic events between China and Europe or the United States. Moreover, many countries in Europe and the United States place great importance on the study of trauma in adolescents and perform types of trauma monitoring to develop effective monitoring systems, such as the American Youth Risk Behavior Surveillance System, the Children’s Hospital damage report and prevention plan, and the Canadian child safety monitoring system [26]. However, there is still no child injury or traumatic event surveillance system in China. Therefore, hospitals, schools, and communities could establish trauma monitoring systems to collect epidemiological data on adolescents who experience traumatic events.

Based on the correlations between parenting style, resilience and post-traumatic symptoms in adolescents shown in Table 3, post-traumatic symptoms are significantly and negatively related to the five subscales of resilience and authoritative parenting style, but they were positively related to authoritarian parenting in all samples. This demonstrated that there is an association between post-traumatic symptoms, parenting style and resilience in adolescents regardless of whether they were experiencing traumatic events that are or are not independent of their parents. In terms of the correlation between the parenting style and resilience, the results in Table 4 show that there are strong associations between “family support” and parenting style, but the correlations between the other subscales of resilience and parenting style cannot be neglected. Therefore, we concluded that the relationships between resilience and parenting style are no more than driven by “family support”.

In Table 5, the family status has a significant negative influence on post-traumatic symptoms, which is in line with a study in Shanghai, China [27]. In the present study, family status is divided into inharmonious and harmonious conditions. We ensured that both inharmonious and harmonious families included both divorced parents and undivided parents because family status can be harmonious even when the parents are divorced. It was indicated that family factors impact adolescents’ post-traumatic symptoms, including negative family factors, such as physical abuse and quarreling, and these can be positively related to post-traumatic symptoms in adolescents [27]. Adolescents who grow up in chronically distressed families are likely to have serious social and emotional problems [15], which impact their ability to address post-traumatic symptoms. Therefore, the harmonious family status is particularly important for the development of mental health in adolescents. Model R_2_ shows that the three different parenting styles had a significant influence on post-traumatic symptoms; the comparison between Models R_2_ and R_1_ shows that the parenting style explains an additional 8.5% of the variance in post-traumatic symptoms. For Model R_3_, the parenting styles and total scores on four of the resilience subscales (not positive thinking) significantly predicted post-traumatic symptoms. The comparison between Models R_3_ and R_2_ shows that resilience explains an additional 9.3% of the variance in post-traumatic symptoms.

Resilience has a negative predictive influence on the post-traumatic symptoms, which is in line with a previous study in south Asia [11]. Individuals who have strong resilience are rich in mental resources, such as life satisfaction, optimism and a tranquil mind. They can invoke psychological resources at any time to cope with difficulties and avoid adversity when they are confronted with a stressful environment. The article has suggested that social support can help individuals recover from PTSD [28], and the subscales of resilience, family support and help-seeking are also types of social support. Moreover, Table 5 shows that parenting style is related to post-traumatic symptoms. Different parenting styles have different effects on adolescents; authoritative parenting is significantly negatively correlated with post-traumatic symptoms in the present study. Under authoritative parenting, children are more likely to form confident, optimistic, and independent personalities, which will help them develop better environmental adaptability, reducing any potential harm in case they experience traumatic events. Adolescents who are exposed to permissive parenting tend to be dependent, irresponsible, selfish and antisocial [29], which can probably explain why permissive parenting has a significant positive correlation with post-traumatic symptoms. A recent study on the association between parenting style and psychopathology in monozygotic twins showed that the perceived differences in parenting style coincide with the differences in levels of major depression, generalized anxiety disorder, conduct disorder and anti-social behavior. This study also showed that differences in authoritarianism were positively related to major depression, generalized anxiety disorder and conduct disorder [30]. This may be helpful in explaining why authoritarian parenting has a positive effect on post-traumatic symptoms.

The SEM of the relationship between the parenting style, resilience, and post-traumatic symptoms is shown in Fig 1, which indicates an integrated association among the three factors. According to the factor-loading parameters and squared multiple correlations of the observed variables shown in the SEM, with the exceptions of permissive parenting and positive thinking, the variable correlations were in the acceptable range. Additionally, the structural parameters of the SEM were significant. Furthermore, the modified goodness-of-fit indexes of the SEM on the data of different samples in Table 1 demonstrated that the association is also suitable for adolescents who went through traumatic events and were or were not independent of their parents and came from either an inharmonious or harmonious family. Therefore, there are strong associations between post-traumatic symptoms, resilience and parenting style, and there is a synthetic, stable association between the parenting style and post-traumatic symptoms, with resilience as a mediator.

The first limitation of this study was that the PAQ scale was modified in a way that might not have been standard and the study only used sections 1 and 3 of the ETI-JK scale. The second was that the participants were chosen only from the Liaoning Province because of time and convenience factors. The third was that the age bracket of the adolescents we investigated was 12–18 years old, which did not fully represent all adolescents in China. The last limitation was that the above three scales did not test the criterion-related validity with corresponding maturity scales. We will conduct future work that is aimed at addressing these issues.

## Conclusions

This paper discussed the relationship between post-traumatic symptoms, parenting style and resilience among adolescents. An authoritative parenting style enhanced adolescents’ resilience and their ability to respond effectively to post-traumatic symptoms, and authoritative parenting should be advocated for Chinese parents. Moreover, schools and society, as a whole, should transfer knowledge on the impact of parenting style to parents and establish and improve adolescents’ regulatory systems to protect and improve their physical and mental security.
